# Women Are Underrepresented Among Authors of Retracted Publications: Retrospective Study of 134 Medical Journals

**DOI:** 10.2196/48529

**Published:** 2023-10-06

**Authors:** Paul Sebo, Joëlle Schwarz, Margaux Achtari, Carole Clair

**Affiliations:** 1 University Institute for Primary Care University of Geneva Geneva Switzerland; 2 Centre for Primary Care and Public Health University of Lausanne Lausanne Switzerland

**Keywords:** error, gender, misconduct, publication, research, retraction, scientific integrity, woman, women, publish, publishing, inequality, retractions, integrity, fraud, plagiarism, research study, research article, scientific research, journal, retrospective

## Abstract

We examined the gender distribution of authors of retracted articles in 134 medical journals across 10 disciplines, compared it with the gender distribution of authors of all published articles, and found that women were underrepresented among authors of retracted articles, and, in particular, of articles retracted for misconduct.

## Introduction

There is extensive literature highlighting the inequalities experienced by female researchers throughout their academic careers [1-3]. By contrast, there is insufficient data on the association between article retractions and gender. A study of 113 PubMed retraction notices from 2016 showed that fraud and plagiarism were found mainly in articles authored by men and errors in data and analysis were seen mainly in articles authored by women [4]. Another study using a database of retracted articles (1970-2022) showed that women represented 27% of first authors and 24% of last authors, but there was no comparison group (ie, the representation of women and men as authors of publications) [5]. There was also no comparison group in a US study that examined 228 cases of misconduct (1994-2012) and found that 149 (65%) were authored by men [6]. Finally, a study assessing factors associated with 611 retractions (2010-2011) found no association with gender, but gender was not determined using a validated tool [7].

In this study, we compared the representation of female first and last authors in retracted articles and all publications by examining 134 medical journals.

## Methods

Multimedia Appendix 1 describes in detail the methods used. For *publications*, we used the results of Hart and Perlis [1], which calculated the proportion of female first and last authors of publications in 134 journals across 10 medical specialties for 2008 and 2017. For *retractions*, we retrieved all PubMed articles published in these journals between January 2003 and December 2022 that were retracted. We evaluated the 2003-2022 period to have a sufficiently large sample size. We retrieved the reason(s) for retraction using the Retraction Watch database and grouped the 102 reasons into 4 main reasons: scientific misconduct only, error(s) only, scientific misconduct and error(s), and reason not related to the author(s). We used the Gender API software to determine first and last authors’ gender [8] and, if inference accuracy was <80%, checked the gender manually by consulting websites with photos. Data extraction was done in duplicate by authors PS and MA. Discrepancies were resolved through discussion among research team members. We assessed first and last authorship as these positions indicate the greatest involvement in the article in most biomedical disciplines.

We computed the proportion of retractions and stratified the results by gender and discipline. To exclude ambiguous names that could skew the gender distribution, we repeated the analyses with retractions whose authors’ gender was determined with >60% or >70% accuracy [1,3]. Data were analyzed descriptively.

Since this study did not involve the collection of personal health-related data, it did not require ethical review, according to current Swiss law.

## Results

**Figure 1:**
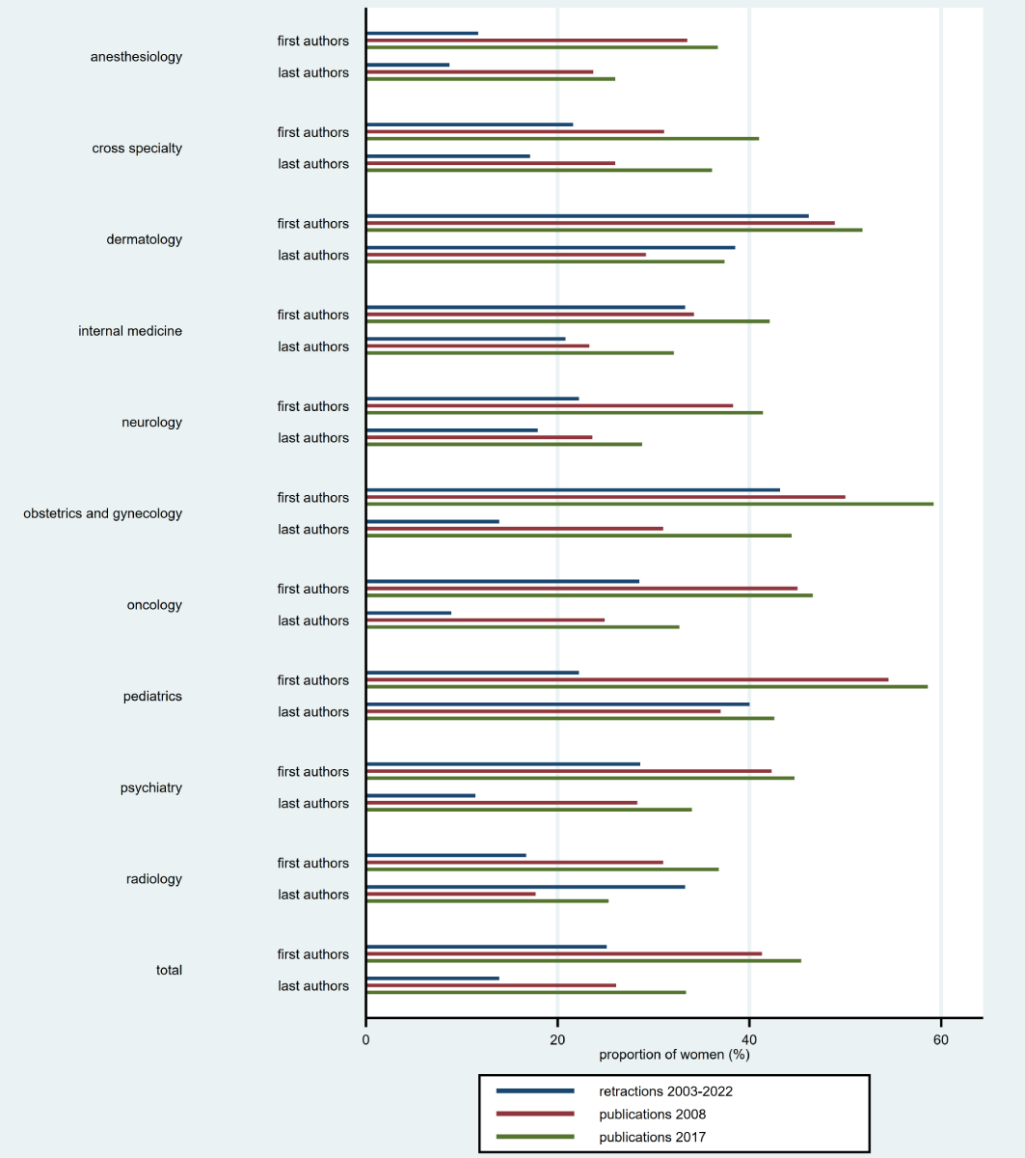
Graph of the proportion of women as first and last authors of retracted articles (2003-2022) and of all publications (2008 and 2017). Data shown by medical specialty.

Table 1 and Figure 1 present the main results. There were 438 retractions for 846,776 articles published between 2003 and 2022 (0.052%). The proportion of retractions was highest in anesthesiology (97/73,458, 0.132%) and lowest in radiology (7/91,162, 0.008%).

After excluding anonymous retracted articles and those with first names as initials, gender could be determined for 398 first authors and 395 last authors. Women were first or last authors of 100 (25.1%) and 55 (13.9%) retractions, respectively, while their proportion as first or last authors of all publications was 41.3% and 26.1% in 2008 and 45.4% and 33.4% in 2017, respectively.

The proportion of female first and last authors of all publications was higher in 2017 than in 2008 for all 10 disciplines. The proportion of women was lower for retractions compared to all publications for all 10 disciplines for first authors and 7 disciplines for last authors.

As shown in Multimedia Appendix 2, compared to men, women were more often first authors of articles retracted for errors (women: 59/115, 51.3%; men: 120/329, 36.5%) and less often for misconduct (women: 53/115, 46.1%; men: 186/329, 56.5%). As last authors, these two reasons were well balanced between women and men.

The results were similar when using subsamples. For example, the proportion of female first and last authors of retractions was 24.3% (93/383) and 13.8% (53/383), respectively, when the authors’ gender was determined with an accuracy of >60%. It was 24.6% (90/366) and 14% (53/379), respectively, when accuracy was >70%.

**Table 1:** Proportion of women as first and last authors of retracted articles (2003-2022) and of all publications (2008 and 2017). Data shown by medical specialty.

Medical specialty^a^	Retractions, n/N (%)	Women as first authors of retractions (2003-2022), n/N (%)	Women as first authors of publications (%)			Women as last authors of retractions (2003-2022), n/N (%)		Women as last authors of publications (%)	
			2008	2017			2008		2017
Anesthesiology	97/73,458 (0.132)	11/94 (11.7)	33.5	36.7	8/92 (8.7)		23.7		26.0
Cross specialty	37/138,754 (0.027)	8/37 (21.6)	31.1	41.0	6/35 (17.1)		26.0		36.1
Dermatology	14/69,631 (0.020)	6/13 (46.2)	48.9	51.8	5/13 (38.5)		29.2		37.4
Internal medicine	26/82,740 (0.031)	8/24 (33.3)	34.2	42.1	5/24 (20.8)		23.3		32.1
Neurology	28/74,315 (0.038)	6/27 (22.2)	38.3	41.4	5/28 (17.9)		23.6		28.8
Obstetrics and gynecology	63/82,144 (0.077)	16/37 (43.2)	50.0	59.2	5/36 (13.9)		31.0		44.4
Oncology	126/123,540 (0.102)	35/123 (28.5)	45.0	46.6	11/123 (8.9)		24.9		32.7
Pediatrics	11/68,924 (0.016)	2/9 (22.2)	54.5	58.6	4/10 (40)		37.0		42.6
Psychiatry	36/60,714 (0.059)	10/35 (28.6)	42.3	44.7	4/35 (11.4)		28.3		34.0
Radiology	7/91,162 (0.008)	1/6 (16.7)	31.0	36.8	2/6 (33.3)		17.7		25.3
Total	438/846,776 (0.052)	100/398 (25.1)	41.3	45.4	55/395 (13.9)		26.1		33.4

^a^The sum of the results of each discipline exceeds the total results because 5 journals were classified into 2 different disciplines.

## Discussion

We found that women were underrepresented among authors of retracted articles, and, in particular, of articles retracted for misconduct.

Compared with the study by Pinho-Gomes et al [5], the proportion of retractions authored by women was similar for first authorship (25% vs 27%) but not for last authorship (14% vs 24%), but these authors included all biomedical journals. Another study showed that women were especially underrepresented among authors of articles retracted for misconduct [4].

Retractions for misconduct can be seen as proxies for scientific integrity, and our results suggest that it varies with gender. Identifying the underlying reasons for these gender disparities is challenging. No studies had directly tackled this topic, making it difficult to draw conclusive findings. Biological, social, and cultural factors can interact in a complex way and contribute to the more pronounced competitive tendencies of men versus women, which can be a possible risk factor for misconduct [9]. Alternatively, women may be less often targeted by investigations than men [10].

Our study has two main limitations. Gender was determined using Gender API and a manual search instead of self-identification. We dichotomized gender into female and male, which did not allow us to assess nonbinary identity.

## Appendix

Multimedia Appendix 1 Methods used in the study.

Multimedia Appendix 2 Reasons for retraction, stratified by gender and authorship.

## Data Availability

The data sets generated and analyzed during this study are available from the corresponding author on reasonable request.
