# Supplementary material for: Women Are Underrepresented Among Authors of Retracted Publications: Retrospective Study of 134 Medical Journals
Source: J Med Internet Res. 2023 Oct 6;25:e48529. doi: 10.2196/48529 (PMC10589828; doi:10.2196/48529)
Supplement: Multimedia Appendix 1 [file jmir_v25i1e48529_app1.docx]

**Multimedia Appendix 1. Methods used in the study**

Detailed description of the methods used in the study:

We used the results of a recent study [1] that calculated for 2008 and 2017 the proportion of women as first/last authors of research articles. The authors included the fifteen journals with the highest impact factor in 2016 for nine medical specialties (anesthesiology, dermatology, internal medicine, neurology, obstetrics/gynecology, oncology, pediatrics, psychiatry, and radiology). They added a tenth category (cross-specialty) consisting of the four most prestigious general medical journals (*New England Journal of Medicine*, *Lancet*, *JAMA* and *BMJ*). The study included 134 not 139 journals because five journals were classified in two different categories. A list of these journals can be found below.

We used the advanced search in PubMed to retrieve all retractions of articles published in these 134 journals between 1 January 2003, i.e. five years before 2008, and 15 December 2022, i.e. five years after 2017 (publication type in PubMed = "retracted publication"). We evaluated the period 2003-2022 (not 2008-2017) to have a sufficiently large sample size. For each retraction, we extracted the name of the first/last author.

We retrieved the reason(s) for retraction using the [Retraction Watch](https://retractionwatch.com/) database. We grouped the 102 reasons in the database into twelve main reasons, as done by other authors before us (*ref: Audisio K, Robinson NB, Soletti GJ, et al. A survey of retractions in the cardiovascular literature. Int J Cardiol. 2022*). After further grouping we obtained the following reasons: scientific misconduct only, error(s) only, scientific misconduct and error(s), and reason not related to the author(s).

We used [Gender API](https://gender-api.com/) to determine the gender of the first/last author. Gender API was found to be accurate in a study comparing several gender detection tools (proportion of misclassifications=1.5%) [8]*.* For authors for whom gender was determined with less than 80% accuracy, we checked the gender manually by consulting websites containing photos.

We computed the proportion of retractions for the years 2003 to 2022 by dividing the number of retractions by the number of articles published in the same journals during the same period. We stratified the results by gender and medical specialty. We repeated the analyses with two subsamples consisting of retractions whose authors' gender was determined with >60% and >70% accuracy, respectively. Data extraction was done in duplicate (PS and MA).

Table #1. Journals included in the study and their impact factor in 2016. Journals shown by medical specialty

| Medical specialty | Journal (NLM title abbreviation) | 2016 Impact factor | NLM ID |
| --- | --- | --- | --- |
| Anesthesiology | Br J Anaesth | 6.24 | 0372541 |
|  | Anesthesiology | 5.79 | 1300217 |
|  | Pain | 5.45 | 7508686 |
|  | Anaesthesia | 4.74 | 0370524 |
|  | J Neurosurg Anesthesiol | 4.03 | 8910749 |
|  | Anesth Analg | 4.01 | 1310650 |
|  | Eur J Anaesthesiol | 3.57 | 8411711 |
|  | Reg Anesth Pain Med | 3.52 | 9804508 |
|  | Clin J Pain | 3.49 | 8507389 |
|  | Eur J Pain | 3.02 | 9801774 |
|  | Pain Physician | 2.84 | 100954394 |
|  | Minerva Anestesiol | 2.62 | 0375272 |
|  | Pain Pract | 2.50 | 101130835 |
|  | Acta Anaesthesiol Scand | 2.44 | 0370270 |
|  | Curr Opin Anaesthesiol | 2.37 | 8813436 |
| Cross Specialty | N Engl J Med | 72.41 | 0255562 |
|  | Lancet | 47.83 | 2985213R |
|  | JAMA | 44.41 | 7501160 |
|  | BMJ | 20.79 | 8900488 |
| Dermatology | J Am Acad Dermatol | 7.00 | 7907132 |
|  | J Invest Dermatol | 6.29 | 0426720 |
|  | JAMA Dermatol | 5.82 | 101589530 |
|  | Pigment Cell Melanoma Res | 5.17 | 101318927 |
|  | Br J Dermatol | 4.71 | 0004041 |
|  | Contact Derm | 4.34 | 7604950 |
|  | J Dermatol Sci | 3.73 | 9011485 |
|  | Acta Dermatovenerol Alp Pannonica Adriat | 3.65 | 9422563 |
|  | J Eur Acad Dermatol Venereol | 3.53 | 9216037 |
|  | Wound Repair Regen | 3.04 | 9310939 |
|  | J Dtsch Dermatol Ges | 2.87 | 101164708 |
|  | Int Wound J | 2.85 | 101230907 |
|  | Skin Pharmacol Physiol | 2.76 | 101188418 |
|  | Am J Clin Dermatol | 2.76 | 100895290 |
|  | Photodermatol Photoimmunol Photomed | 2.66 | 9013641 |
| Internal Medicine | Ann Intern Med | 17.20 | 0372351 |
|  | JAMA Intern Med | 16.54 | 101589534 |
|  | PLoS Med | 11.86 | 101231360 |
|  | J Cachexia Sarcopenia Muscle | 9.70 | 101552883 |
|  | BMC Med | 8.10 | 101190723 |
|  | J Intern Med | 7.98 | 8904841 |
|  | Mayo Clin Proc | 6.86 | 0405543 |
|  | CMAJ | 6.78 | 9711805 |
|  | Nat Rev Dis Primers | 6.39 | 101672103 |
|  | Cochrane Database Syst Rev | 6.26 | 100909747 |
|  | Am J Med | 5.55 | 0267200 |
|  | Ann Fam Med | 4.92 | 101167762 |
|  | Transl Res | 4.65 | 101280339 |
|  | Dtsch Arztebl Int | 4.26 | 101475967 |
|  | Palliat Med | 4.22 | 8704926 |
| Neurology | Lancet Neurol | 26.28 | 101139309 |
|  | Nat Rev Neurol | 20.26 | 101500072 |
|  | Acta Neuropathol | 12.21 | 0412041 |
|  | Brain | 10.29 | 0372537 |
|  | JAMA Neurol | 10.03 | 101589536 |
|  | Ann Neurol | 9.89 | 7707449 |
|  | Alzheimers Dement | 9.48 | 101231978 |
|  | Sleep Med Rev | 8.96 | 9804678 |
|  | Neurology | 8.32 | 0401060 |
|  | Neuro Oncol | 7.79 | 100887420 |
|  | Neuroscientist | 7.39 | 9504819 |
|  | J Neurol Neurosurg Psychiatry | 7.35 | 2985191R |
|  | Mov Disord | 7.07 | 8610688 |
|  | Brain Pathol | 6.62 | 9216781 |
|  | Alzheimers Res Ther | 6.15 | 101511643 |
| Obstetrics and Gynecology | Hum Reprod Update | 11.75 | 9507614 |
|  | Am J Obstet Gynecol | 5.23 | 0370476 |
|  | Obstet Gynecol | 5.22 | 0401101 |
|  | BJOG | 5.05 | 100935741 |
|  | Hum Reprod | 5.02 | 8701199 |
|  | Gynecol Oncol | 4.96 | 0365304 |
|  | Ultrasound Obstet Gynecol | 4.71 | 9108340 |
|  | Fertil Steril | 4.45 | 0372772 |
|  | Pregnancy Hypertens | 3.93 | 101552483 |
|  | Mol Hum Reprod | 3.59 | 9513710 |
|  | Maturitas | 3.26 | 7807333 |
|  | Reprod Biomed Online | 3.25 | 101122473 |
|  | Clin Perinatol | 3.23 | 7501306 |
|  | Semin Perinatol | 3.19 | 7801132 |
|  | J Gynecol Oncol | 3.14 | 101483150 |
| Oncology | CA Cancer J Clin | 187.04 | 0370647 |
|  | Nat Rev Cancer | 37.15 | 101124168 |
|  | Lancet Oncol | 33.90 | 100957246 |
|  | Cancer Cell | 27.41 | 101130617 |
|  | J Clin Oncol | 24.01 | 8309333 |
|  | Nat Rev Clin Oncol | 20.69 | 101500077 |
|  | Cancer Discov | 20.01 | 101561693 |
|  | JAMA Oncol | 16.56 | 101652861 |
|  | J Natl Cancer Inst | 12.59 | 7503089 |
|  | Ann Oncol | 11.86 | 9007735 |
|  | Leukemia | 11.70 | 8704895 |
|  | Clin Cancer Res | 9.62 | 9502500 |
|  | Biochim Biophys Acta | 9.45 | 0217513 |
|  | Semin Cancer Biol | 9.14 | 9010218 |
|  | Cancer Res | 9.12 | 2984705R |
| Pediatrics | JAMA Pediatr | 10.25 | 101589544 |
|  | J Am Acad Child Adolesc Psychiatry | 6.44 | 8704565 |
|  | Pediatrics | 5.71 | 0376422 |
|  | Pediatr Diabetes | 4.27 | 100939345 |
|  | Arch Dis Child Fetal Neonatal Ed | 4.10 | 9501297 |
|  | J Adolesc Health | 3.97 | 9102136 |
|  | J Pediatr | 3.87 | 0375410 |
|  | Pediatr Allergy Immunol | 3.78 | 9106718 |
|  | Pediatr Crit Care Med | 3.50 | 100954653 |
|  | Pediatr Obes | 3.40 | 101572033 |
|  | Semin Fetal Neonatal Med | 3.33 | 101240003 |
|  | Eur Child Adolesc Psychiatry | 3.30 | 9212296 |
|  | Arch Dis Child | 3.27 | 0372434 |
|  | Clin Perinatol | 3.23 | 7501306 |
|  | Semin Perinatol | 3.19 | 7801132 |
| Psychiatry | World Psychiatry | 26.56 | 101189643 |
|  | JAMA Psychiatry | 15.31 | 101589550 |
|  | Am J Psychiatry | 14.18 | 0370512 |
|  | Mol Psychiatry | 13.20 | 9607835 |
|  | Lancet Psychiatry | 11.59 | 101638123 |
|  | Biol Psychiatry | 11.41 | 0213264 |
|  | Psychother Psychosom | 8.96 | 0024046 |
|  | Schizophr Bull | 7.58 | 0236760 |
|  | J Neurol Neurosurg Psychiatry | 7.35 | 2985191R |
|  | Acta Psychiatr Scand | 6.79 | 0370364 |
|  | Neuropsychopharmacology | 6.40 | 8904907 |
|  | J Am Acad Child Adolesc Psychiatry | 6.44 | 8704565 |
|  | Br J Psychiatry | 6.35 | 0342367 |
|  | J Child Psychol Psychiatry | 6.23 | 0375361 |
|  | Addiction | 5.79 | 9304118 |
| Radiology, Nuclear Medicine, and Imaging | JACC Cardiovasc Imaging | 10.19 | 101467978 |
|  | Radiology | 7.30 | 0401260 |
|  | Eur J Nucl Med Mol Imaging | 7.28 | 101140988 |
|  | Circ Cardiovasc Imaging | 6.80 | 101479935 |
|  | J Nucl Med | 6.65 | 0217410 |
|  | Neuroimage | 5.84 | 9215515 |
|  | J Cardiovasc Magn Reson | 5.60 | 9815616 |
|  | Semin Radiat Oncol | 5.36 | 9202882 |
|  | Invest Radiol | 5.20 | 0045377 |
|  | Int J Radiat Oncol Biol Phys | 5.13 | 7603616 |
|  | Ultrasound Obstet Gynecol | 4.71 | 9108340 |
|  | Clin Nucl Med | 4.56 | 7611109 |
|  | Hum Brain Mapp | 4.53 | 9419065 |
|  | Radiother Oncol | 4.33 | 8407192 |
|  | Med Image Anal | 4.19 | 9713490 |

Table #2. Description of the 102 reasons for retraction in Retraction Watch Database, and grouping into twelve and five main reasons.

| Code for main reason (N=12) | Reason (N=102) | Description |
| --- | --- | --- |
| BEHA | Author Unresponsive | Author(s) lack of communication after prior contact by Journal, Publisher or other original Authors |
| ERRO | Bias Issues or Lack of Balance | Failure to maintain objectivity in the presentation or analysis of information |
| BEHA | Breach of Policy by Author | A violation of the Journal, Publisher or Institutional accepted practices by the author |
| NA | Breach of Policy by Third Party | A violation of the Journal, Publisher or Institutional accepted practices by a person or company/institution not the authors |
| CITE | Cites Retracted Work | A retracted item is used in citations or referencing |
| NA | Civil Proceedings | Non-criminal litigation arising from the publication of the original article or the related notice(s) |
| NA | Complaints about Author | Allegations made strictly about the author without respect to the original article |
| NA | Complaints about Company/Institution | Allegations made strictly about the author’s affiliation(s) without respect to the original article |
| NA | Complaints about Third Party | Allegations made strictly about those not the author or the author’s affiliation(s) without respect to the original article |
| AUTH | Concerns/Issues About Authorship | Any question, controversy or dispute over the rightful claim to authorship, excluding forged authorship |
| ERRO | Concerns/Issues About Data | Any question, controversy or dispute over the validity of the data |
| ERRO | Concerns/Issues About Image | Any question, controversy or dispute over the validity of the image |
| ERRO | Concerns/Issues about Referencing/Attributions | Any question, controversy or dispute over whether ideas, analyses, text or data are properly credited to the originator |
| ERRO | Concerns/Issues About Results | Any question, controversy or dispute over the validity of the results |
| NA | Concerns/Issues about Third Party Involvement | Any question, controversy or dispute over the rightful claim to authorship, excluding forged authorship |
| CONF | Conflict of Interest | Authors having affiliations with companies, associations, or institutions that may serve to influence their belief about their findings |
| ERRO | Contamination of Cell Lines/Tissues | Impurities found within cell lines or tissues |
| ERRO | Contamination of Materials (General) | Impurities found within compounds or solutions used in experiments |
| ERRO | Contamination of Reagents | Impurities found within compounds or solutions used to drive experimental outcomes |
| NA | Copyright Claims | Dispute concerning right of ownership of a publication |
| NA | Criminal Proceedings | Court actions that may result in incarceration or fines arising from the publication of the original article or the related notice(s) |
| NA | Date of Retraction/Other Unknown | A lack of publishing date given on the notice – or the date on the notice is not representative of the actual notice date. Commonly found when Publishers overwrite the original article’s HTML page with the retraction notice, without changing the publication date to reflect such. |
| NA | Doing the Right Thing | An attribution made by co-founders of Retraction Watch indicating admirable behavior by one of the involved parties |
| DUPL | Duplication of Article | Also known as “self-plagiarism”. Used when an entire published item, or undefined sections of it, written by one or all authors of the original article, are repeated in the original article without appropriate citation. |
| DUPL | Duplication of Data | Also known as “self-plagiarism”. Used when the all or part of the data from an item written by one or all authors of the original article, are repeated in the original article without appropriate citation. |
| DUPL | Duplication of Image | Also known as “self-plagiarism”. Used when an image from an item written by one or all authors of the original article is repeated in the original article without appropriate citation. |
| DUPL | Duplication of Text | Also known as “self-plagiarism”. Used when sections of text from an item written by one or all authors of the original article, are repeated in the original article without appropriate citation. |
| NA | Duplicate Publication through Error by Journal/Publisher | Used when a Journal or Publisher incorrectly publishes the same article more than once. Differs from Duplication of Article, which is typically due to dual submission by the article’s authors. |
| NA | Error by Journal/Publisher | A mistake attributed to a Journal Editor or Publisher |
| NA | Error by Third Party | A mistake attributed to a person or other, who is not an author or representative of the Journal or Publisher |
| ERRO | Error in Analyses | A mistake made in the evaluation of the data or calculations |
| ERRO | Error in Cell Lines/Tissues | A mistake made in the identification of cell lines or tissues, or the choice of an incorrect cell line or tissue |
| ERRO | Error in Data | A mistake made in the data, either in data entry, gathering or identification |
| ERRO | Error in Image | A mistake made in the preparation or printing of an image |
| ERRO | Error in Materials (General) | A mistake made in the choice of materials in the performance of experiments (eg., reagents, mixing bowls, etc) |
| ERRO | Error in Methods | A mistake made in the experimental protocol, either in following the wrong protocol, or in erring during the performance of the protocol |
| ERRO | Error in Results and/or Conclusions | A mistake made in determining the results or establishing conclusions from an experiment or analysis |
| ERRO | Error in Text | A mistake made in the written portion of the item |
| ETHI | Ethical Violations by Author | When an author performs an action contrary to accepted standards of behavior. Generally used only when stated as such in the notice and no other specific reason (e.g., duplication of image) is given. |
| NA | Ethical Violations by Third Party | When any person not an author performs an action contrary to accepted standards of behavior. Generally used only when stated as such in the notice and no other specific reason (e.g., duplication of image) is given. |
| NA | Euphemisms for Duplication | The notice does not clearly state that the authors reused ideas, text, or images from one of their previously published items without suitable citation |
| NA | Euphemisms for Misconduct | The notice does not clearly state that the reason for the notice is due to fabrication, falsification, or plagiarism by one or all the authors, despite an institutional report stating such. |
| NA | Euphemisms for Plagiarism | The notice does not clearly state that the authors reused ideas, text, or images, without suitable citation, from items published by those not the authors |
| NA | Fake Peer Review | The peer review was intentionally not performed in accordance with the journal’s guidelines or ethical standards |
| FALS | Falsification/Fabrication of Data | Intentional changes to data so that it is not representative of the actual finding |
| FALS | Falsification/Fabrication of Image | Intentional changes to an image so that it is not representative of the actual data |
| FALS | Falsification/Fabrication of Results | Intentional changes to results so that it is not representative of the actual finding |
| AUTH | Forged Authorship | The fraudulent use of an author name in submitting a manuscript for publication |
| NA | Hoax Paper | Paper intentionally drafted with fraudulent data or information with the specific intent of testing a journal’s or publisher’s manuscript acceptance policies |
| ETHI | Informed/Patient Consent – None/Withdrawn | When the full risks and benefits from being in an experiment are not provided to and accepted by the participant, or the participant chooses to later recant their approval |
| NA | Investigation by Company/Institution | An evaluation of allegations by the affiliations of one or all of the authors |
| NA | Investigation by Journal/Publisher | An evaluation of allegations by the Journal or Publisher |
| NA | Investigation by ORI | An evaluation of allegations by the United State Office of Research Integrity |
| NA | Investigation by Third Party | An evaluation of allegations by a person, company or institution not the Authors, Journal, Publisher or ORI |
| OTHE | Lack of Approval from Author | Failure to obtain agreement from original author(s) |
| NA | Lack of Approval from Company/Institution | Failure to obtain agreement from original author(s) |
| NA | Lack of Approval from Third Party | Failure to obtain agreement from original author(s) |
| ETHI | Lack of IRB/IACUC Approval | Failure to obtain consent from the institutional ethical review board overseeing human or animal experimentation prior to initiation of study, or failure to provide proof of such |
| NA | Legal Reasons/Legal Threats | Actions taken to avoid or foster litigation |
| FALS | Manipulation of Images | The changing of the presentation of an image by reversal, rotation or similar action |
| FALS | Manipulation of Results | The changing of the presentation of results which may lead to conclusions not otherwise warranted |
| BEHA | Miscommunication by Author | Error in messaging from or to author |
| NA | Miscommunication by Company/Institution | Error in messaging from or to authors’ affiliations |
| NA | Miscommunication by Journal/Publisher | Error in messaging from or to Journal or Publisher |
| NA | Miscommunication by Third Party | Error in messaging from or to any party not the author, affiliations, journal of publisher |
| NA | Misconduct – Official Investigation/Finding | Finding of misconduct after investigation by incorporated company, institution of governmental agency |
| UNSP | Misconduct by Author | Statement Journal, Publisher, Company, Institution, Governmental Agency, or Author that author committed misconduct |
| NA | Misconduct by Company/Institution | Statement Journal, Publisher, Company, Institution, or Governmental Agency that Company/Institution committed misconduct |
| NA | Misconduct by Third Party | Statement Journal, Publisher, Company, Institution, or Governmental Agency that a third party committed misconduct |
| NA | No Further Action | Generally applicable to Expressions of Concern – Statement by Editor or Publisher that |
| BEHA | Nonpayment of Fees/Refusal to Pay | Lack of payment of full amount due for services rendered or for rights of access |
| NA | Notice – Lack of | No Notice was published by the Journal or Publisher, and the article is removed from the publishing platform. |
| NA | Notice – Limited or No Information | A notice provides minimal information as to the cause of the notice, or the original item is watermaked as retracted or corrected without explanation |
| NA | Notice – Unable to Access via current resources | The notice is paywalled, only in print, or in some form unavailable for inspection at this time. |
| NA | Objections by Author(s) | A complaint by any of the original authors or refusal to agree actions taken by the Journal or Publisher |
| NA | Objections by Company/Institution | A complaint by any of the original authors’ affiliation(s) or refusal by same to agree actions taken by the Journal or Publisher |
| NA | Objections by Third Party | A complaint by any person, company or institution not of the original authors, or refusal by same to agree actions taken by the Journal or Publisher |
| BEHA | Original Data not Provided | The original data or images for the published study is no longer available or is not given to the editorial staff. |
| FALS | Paper Mill | A business that creates and sells research papers where the buyer(s) claim authorship; the business is generally unacknowledged as a contributor. |
| PLAG | Plagiarism of Article | Used when an entire published item, or undefined sections of it, and not written by one or all authors of the original article, are repeated in the original article without appropriate citation. |
| PLAG | Plagiarism of Data | Used when the all or part of the data from an item not written by one or all authors of the original article, are repeated in the original article without appropriate citation. |
| PLAG | Plagiarism of Image | Used when an image from an item not written by one or all authors of the original article is repeated in the original article without appropriate citation. |
| PLAG | Plagiarism of Text | Used when sections of text from an item not written by one or all authors of the original article, are repeated in the original article without appropriate citation. |
| NA | Publishing Ban | A Journal or Publisher states that no manuscripts will be acceptance from one or all the authors of the original article. It can be for a limited time, or indefinitely. |
| FALS | Randomly Generated Content | Text or data that was created via a randomizing algorithm such as Mathgen or Scigen |
| NA | Results Not Reproducible | Experiments conducted, using the same materials and methods, that fail to replicate the finding of the original article |
| NA | Retract and Replace | The permanent change of an item to a non-citable status, with a subsequent republication by the same journal after substantial changes to the item |
| NA | Rogue Editor | Applies to fraudulent editor (i.e. non-existent or with false credential); applies to those lists as “Guest Editor” as well; |
| OTHE | Sabotage of Materials | An intentional action to surreptitiously change or contaminate experimental ingredients in order to artificially change the experimental outcome |
| OTHE | Sabotage of Methods | An intentional action to surreptitiously change or contaminate experimental instruments or tools in order to artificially change the experimental outcome |
| OTHE | Salami Slicing | The publication of several articles by using the same (small) dataset, but by breaking it into sections, with the intent of exploiting a limited data set for the production of several published works This does not apply to large multi-group studies such as the Framingham Heart Study. |
| NA | Temporary Removal | An original article is removed from the Journal’s publishing platform for an undefined period of time, after which, if returned to the publishing platform, it appears with minimal or no substantial changes |
| NA | Transfer of Copyright/Ownership | Articles removed from a publishing platform due to change in the Copyright/Ownership of the article |
| ERRO | Unreliable Data | The accuracy or validity of the data is questionable |
| ERRO | Unreliable Image | The accuracy or validity of the image is questionable |
| ERRO | Unreliable Results | The accuracy or validity of the results is questionable |
| ERRO | Updated to Correction | A prior notice has been changed to the status of a Correction |
| NA | Updated to Retraction | A prior notice has been changed to the status of a Retraction |
| NA | Upgrade/Update of Prior Notice | Either a change to or affirmation of a prior notice |
| NA | Withdrawal | The original article is removed from access on the Journal’s publishing platform. |
| NA | Withdrawn (out of date) | The article has been retracted as part of the journal’s normal process of keeping guidelines or reviews current for professional use. |
| NA | Withdrawn to Publish in Different Journal | Journal/Publisher removed an article from one journal platform to publish in a different journal platform. |

Code and description of the twelve main raisons:

BEHA: issue(s) with authors (any controversy about the behavior of one or more authors)

ERRO: error(s) within the manuscript (data, images, methods, results, referencing, text, materials)

NA: reason not related to the author(s)

CITE: cites prior retracted work

AUTH: issue(s) about authorship

CONF: conflict of interest

DUPL: duplication (article, data, image, text)

ETHI: ethical violation(s)

FALS: falsification or fabrication (data, images, results)

OTHE: other reason (scientific misconduct)

UNSP: reason not specified

PLAG: plagiarism (article, data, image, text)

Grouping into five main reasons:

Scientific misconduct: BEHA + AUTH + CONF + DUPL + ETHI + FALS + OTHE + PLAG

Error(s): ERRO + CITE

Reason not specified: UNSP (number of retractions = 0)

Reason not related to the author(s): NA

Note that, as there were no observations for UNSP alone, the grouping led to four and not five main reasons.
