# Supplementary material for: Women Are Underrepresented Among Authors of Retracted Publications: Retrospective Study of 134 Medical Journals
Source: J Med Internet Res. 2023 Oct 6;25:e48529. doi: 10.2196/48529 (PMC10589828; doi:10.2196/48529)
Supplement: Multimedia Appendix 2 [file jmir_v25i1e48529_app2.docx]

**Multimedia Appendix 2. Reasons for retraction, stratified by gender and authorship**

| Reason for retraction^1^ | Women as first authors, n/N (%) | Men as first authors, n/N (%) | Women as last authors, n/N (%) | Men as last authors, n/N (%) |
| --- | --- | --- | --- | --- |
| Scientific misconduct only | 32 / 94 (34.0) | 147 / 290 (50.7) | 26 / 51 (51.0) | 153 / 330 (46.4) |
| *[ Scientific misconduct +/- error(s) ]* | *[ 53 / 115 (46.1) ]* | *[ 186 / 329 (56.5) ]* | *[ 31 / 56 (55.4) ]* | *[ 208 / 385 (54.0) ]* |
| Error(s) only | 38 / 94 (40.5) | 81 / 290 (27.9) | 19 / 51 (37.2) | 99 / 330 (30.0) |
| *[ Error(s) +/- scientific misconduct ]* | *[ 59 / 115 (51.3) ]* | *[ 120 / 329 (36.5) ]* | *[ 24 / 56 (42.9) ]* | *[154 / 385 (40.0) ]* |
| Scientific misconduct and error(s) | 21 / 94 (22.3) | 39 / 290 (13.5) | 5 / 51 (9.8) | 55 / 330 (16.6) |
| Reason not related to the author(s) | 3 / 94 (3.2) | 23 / 290 (7.9) | 1 / 51 (2.0) | 23 / 330 (7.0) |

^1^Missing data =14 for both first and last authorship
